# Integrated ultrasonographic, endocrine, seminal, and morphological characterization of cryptorchidism in collared peccary (*Pecari tajacu*)

**DOI:** 10.1590/1984-3143-AR2026-0022

**Published:** 2026-07-20

**Authors:** Radan Elvis Matias de Oliveira, Fernanda Loffler Niemeyer Attademo, Alana Ingrid de Araújo Pereira, Euziele Oliveira de Santana, Romário Parente dos Santos, Luana Grasiele Pereira Bezerra, Alexandre Rodrigues Silva, Moacir Franco de Oliveira

**Affiliations:** 1 Laboratório de Morfofisiologia Animal Aplicada, Universidade Federal Rural do Semi-Árido, Mossoró, RN, Brasil; 2 Programa de Pós-graduação em Ciência Animal, Universidade Federal da Paraíba, Areia, PB, Brasil; 3 Laboratório de Conservação de Germoplasma Animal, Universidade Federal Rural do Semi-Árido, Mossoró, RN, Brasil

**Keywords:** andrological evaluation, reproductive anomalies, reproductive diagnosis, Tayassuidae

## Abstract

Cryptorchidism is a congenital reproductive disorder that can compromise fertility and represents a challenge for conservation and management programs in wildlife species. In collared peccary (*Pecari tajacu*), reports of this condition are rare and generally limited to imaging findings. This study reports a case of unilateral cryptorchidism in an adult male collared peccary, providing an integrated ultrasonographic, biometric, endocrine, seminal, and morphological characterization. Clinical examination revealed the absence of the left testis in the scrotum, with an inguinal mass suggestive of testicular retention, which was confirmed by ultrasonography. Ultrasonographic evaluation demonstrated a retained testis with reduced size and homogeneous hypoechoic echotexture, whereas the scrotal testis showed increased volume and discrete echotextural alterations. Testicular biometry revealed marked asymmetry between the testes, with reduced length, width, thickness, and volume of the cryptorchid testis compared with the contralateral scrotal testis, which exhibited hypertrophy. Serum testosterone concentration was markedly elevated (15,940 pg/mL), exceeding reference values for the species. Seminal analysis revealed reduced sperm concentration (380 × 10^6^ spermatozoa/mL) compared to healthy individuals, although motility (74%) and sperm morphology (69% normal cells) remained within ranges compatible with fertility. Reduced mitochondrial activity and plasma membrane functionality suggested potential limitations in functional fertility. Histological evaluation demonstrated preserved architecture and complete spermatogenesis in the scrotal testis, whereas the retained testis exhibited tubular atrophy and absence of advanced stages of spermatogenesis. Despite methodological considerations regarding hormonal assessment, the findings support a compensatory hypertrophic and endocrine response of the scrotal testis in unilateral cryptorchidism. This case expands current knowledge on reproductive pathology in collared peccary and highlights the importance of integrated diagnostic approaches for reproductive health assessment and management in captive wildlife populations.

## Introduction

The collared peccary (*Pecari tajacu*) is a wild mammal belonging to the family Tayassuidae and is widely distributed throughout Central and South America (Sowls, 1997; Gongora et al., 2011). This species plays an important ecological role in the ecosystems it inhabits, contributing to seed dispersal and the maintenance of environmental balance (Desbiez et al., 2012; Pérez-Irineo and Santos-Moreno, 2016). In addition to its ecological relevance, the collared peccary has zootechnical potential and is raised in production systems aimed at both conservation and the sustainable use of its products (Morais et al., 2022).

The study of reproductive pathologies in wild species is essential, as conditions that compromise fertility can affect not only reproduction in captivity but also *ex situ* and *in situ* conservation strategies (Comizzoli and Holt, 2019). Among these conditions, cryptorchidism deserves particular attention, as it can reduce individual reproductive potential and compromise management and assisted reproduction programs in threatened animals (Qian et al., 2024).

Cryptorchidism is characterized by the failure of testicular migration to the scrotal sac during embryonic or postnatal development and is mainly associated with genetic factors (Chai et al., 2021; Pei et al., 2025). This anomaly is widely described in domestic species (Han et al., 2020; Pecile et al., 2021; Bhaskaran et al., 2025; Pei et al., 2025), in which it is associated with infertility, testicular degeneration, hormonal alterations (Rodprasert et al., 2020; Hernández-Jardón et al., 2022), and an increased predisposition to the development of testicular neoplasms (Chai et al., 2021; Pecile et al., 2021). In contrast, in wild species, particularly in collared peccary, reports remain scarce, with only a single case of unilateral cryptorchidism described, diagnosed exclusively by ultrasonographic examination (Peixoto et al., 2010), without complementary hormonal, seminal, or histological evaluation.

In this context, the present study is justified by the rarity of cryptorchidism diagnoses in collared peccaries and by the need to expand the understanding of the reproductive repercussions of this condition in the species. Thus, this study aims to report a case of cryptorchidism in collared peccary, providing an integrated description of ultrasonographic, hormonal, seminal, and morphological findings, thereby contributing to knowledge of the reproductive health of this wild species and supporting future strategies for management, conservation, and assisted reproduction.

## Methods

### Ethics statement

Ethical approval by an Animal Research Ethics Committee is not required for this type of study.

### Animal history

A collared peccary, adult male, two years old and weighing approximately 23 kg, born in captivity at the Center for the Multiplication of Wild Animals of the Federal Rural University of the Semi-Arid (CEMAS/UFERSA), located in Mossoró, Rio Grande do Norte, Brazil (5°12′49.4″S; 37°18′36.7″W), was evaluated in August 2025 during routine management procedures. The animal was housed in a collective enclosure with other adult males, with no history of aggressive behavior or evident hierarchical disputes. The individual had never mated, nor had there been any attempt at reproductive contact with females. The daily diet consisted of fruits, corn grains, commercial feed, and water *ad libitum*.

### Clinical examination

**Figure 1:**
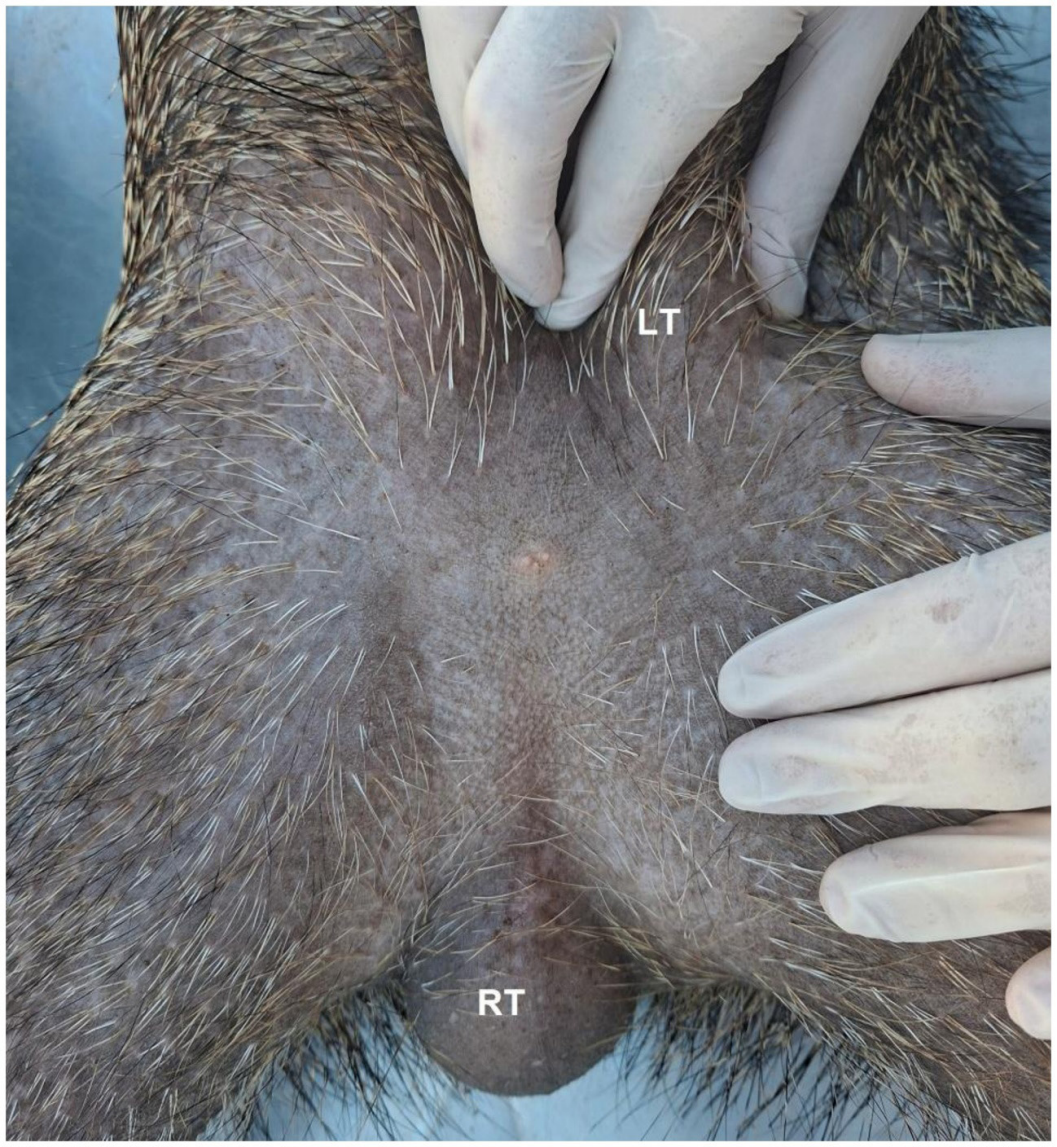
Physical examination of a collared peccary (*Pecari tajacu*) with unilateral cryptorchidism. On palpation of the left inguinal region, an increased volume consistent with the presence of a retained left testicle (LT) was observed, while the right testicle (RT) was properly positioned within the scrotal sac.

During the clinical examination, performed after physical restraint using a capture net, it was observed that the animal presented only one testicle within the scrotal sac, while the other testicle was not identified in its usual anatomical position. Subsequently, the animal was anesthetized for physical examination using propofol (Propovan®, Cristália, Fortaleza, Brazil) at an intravenous dose of 5 mg/kg, with maintenance by continuous bolus infusion of the same drug. On palpation of the left inguinal region, an increase in volume consistent with the presence of a retained testicle was detected (Figure 1).

### Ultrasonographic examination

Under anesthesia, with the animal in dorsal recumbency, an ultrasonographic examination was performed to characterize testicular biometry, echotexture, and echogenicity using a portable Mindray Z50^®^ ultrasound system (Shenzhen Mindray Bio-Medical Electronics Co., Shenzhen, China) equipped with a 7–12 MHz linear transducer. Measurements of testicular length, width, and thickness followed the methodology described by Peixoto et al. (2016) and were obtained using electronic calipers integrated into the ultrasound system. The calipers were positioned at the boundaries of the tunica albuginea. At least three distinct images in the sagittal and transverse planes were acquired for each testicle, and the largest measurement was recorded for each testicular dimension. Care was taken to avoid inclusion of the epididymis during testicular measurements.

### Testosterone analysis

For serum testosterone analysis, blood was collected by venipuncture of the saphenous vein and placed into 3 mL tubes containing a clot activator (Vacuplast^®^). After collection, the tubes were sent to the laboratory and centrifuged at 2,000–3,000 × g for 10 minutes to separate the serum, which was then aliquoted into 1.5 mL microtubes and stored at −20 °C until hormonal analysis. Serum testosterone concentration was determined according to the methodology described by Maia et al. (2019), using liquid-phase radioimmunoassay (RIA) with a commercial kit (RIA Testosterone, Beckman Coulter, Brea, CA, USA), in a Wizard system detector (PerkinElmer do Brasil Ltda.). Results were expressed in pg/mL.

### Semen analysis

While still under anesthesia, the animal underwent semen collection in lateral recumbency by electroejaculation, following a protocol previously described for the species (Castelo et al., 2010). A portable device (Autojac®, Neovet, Campinas, SP, Brazil) connected to a 12 V power source was used, to which a rectal probe measuring 15 cm in length and 1.3 cm in diameter was attached. The probe was inserted approximately 12 cm into the animal’s rectum. The semen was collected in plastic tubes and immediately evaluated.

Semen volume was measured using micropipettes. Sperm concentration (in millions of spermatozoa/mL) was determined using a Neubauer counting chamber. Based on sperm concentration and semen volume, the total number of spermatozoa per ejaculate was calculated (Peixoto et al., 2012).

Sperm motility kinematic patterns were determined by computer-assisted analysis (IVOS 7.4G, Hamilton-Thorne Research™, Beverly, MA, USA), according to settings previously established for the species (Souza et al., 2015). The following parameters were evaluated: total motility (%), progressive motility (%), average path velocity (VAP; μm/s), straight-line velocity (VSL; μm/s), curvilinear velocity (VCL; μm/s), amplitude of lateral head displacement (ALH; μm), beat-cross frequency (BCF; Hz), straightness (STR; %), and linearity (LIN; %), as well as sperm subpopulations classified as rapid, medium, slow, and static.

For the analysis of sperm plasma membrane integrity, a fluorescent solution of carboxyfluorescein diacetate (CFDA) and propidium iodide (PI) was used. Samples were incubated for 10 min at 27 °C in the CFDA + PI solution and subsequently evaluated by epifluorescence microscopy (episcopic fluorescent halogen lamp system “EFA”; Leica, Kista, Sweden). For each sample, 200 spermatozoa were counted, and those entirely stained green (CFDA) were classified as having intact membranes, whereas those totally or partially stained red (PI) were considered to have non-intact membranes (Sousa et al., 2013). In addition, cells showing red staining in the midpiece were considered to exhibit mitochondrial activity (Moreira et al., 2024).

Membrane functionality was investigated using the hypo-osmotic swelling test, with distilled water (0 mOsm/L) as the hypo-osmotic solution. A semen aliquot was diluted (1:9) and incubated at 37 °C for 45 minutes. Subsequently, a total of 200 cells were counted under phase-contrast microscopy (400×; Alttion®, Wuzhou City, Guangxi Province, China) and analyzed for osmotic response, with cells exhibiting tail curling considered to have functional membranes (Moreira et al., 2024).

For sperm morphology analysis (%), Rose Bengal–stained smears were evaluated by light microscopy (1000×), with 200 cells counted per slide and classified according to the presence or absence of morphological defects (Moreira et al., 2024).

### Morphological analysis of the testes

At the end of the procedures, considering that the maintenance of cryptorchid animals is not recommended in captive breeding populations for reproductive reasons, since this condition is often associated with genetic inheritance and may compromise fertility (Fan et al., 2021), euthanasia of the individual was performed in accordance with current ethical and animal welfare guidelines (CFMV, 2012). Initially, a combination of xylazine hydrochloride (2 mg/kg) and ketamine hydrochloride (20 mg/kg), both administered intramuscularly, was used as preanesthetic medication. Propofol (5 mg/kg) was administered intravenously to induce anesthesia. After achieving the desired anesthetic plane, potassium chloride (1 mL/kg) was administered intravenously to perform euthanasia.

After euthanasia, the testes were dissected and evaluated *in situ*, then removed and analyzed *ex situ* with respect to shape and coloration, and subsequently subjected to biometric and histological evaluation. Testicular biometry was assessed by measuring the length (L), width (W), and thickness (T) of each testis, as described by Sonner et al. (2004). These measurements were used to calculate testicular volume (V) using Lambert’s formula (Lambert, 1951): V = L × T × W × 0.71. In addition, each testis was individually weighed using a precision balance.

After biometric evaluation, the testes were fixed in 4% paraformaldehyde and subsequently processed for histological analysis, following the steps described by Tolosa et al. (2003). Histological sections (~5 µm) were obtained using a LEICA RM2125 RT® microtome, mounted on glass slides, and kept in an oven at 60 °C overnight. The slides were stained with hematoxylin and eosin (H&E) for histological evaluation. Analyses were performed using a LEICA DM500 HD light microscope coupled to a LEICA ICC50W camera, and images were acquired using LAS EZ Ink software. The histological terminology used follows the recommendations of the International Committee on Veterinary Histological Nomenclature (2017).

## Results

### Ultrasonography

**Figure 2:**
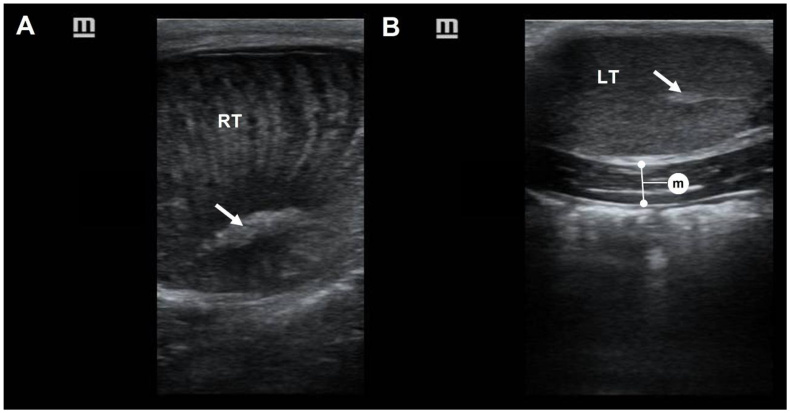
Ultrasonographic findings of the testes of a collared peccary (*Pecari tajacu*) with unilateral cryptorchidism. (A) Right testis, located within the scrotal sac, showing preserved contour, shape, and mediastinal line, and a mixed echotexture, with echogenic lines alternating with hypoechogenic areas distributed throughout the testicular parenchyma and converging toward the hyperechogenic mediastinum; (B) Left testis, located in the inguinal region, in the subcutaneous tissue, showing preserved contour and shape, decreased echogenicity, and homogeneous echotexture, with a central hyperechogenic mediastinum. Legend: right testis (RT); left testis (LT); testicular mediastinum (arrow); abdominal wall muscles (m).

On ultrasonographic examination, the right testis was located within the scrotal sac and showed a markedly increased size (length: 6.3 cm; width: 4.3 cm; thickness: 3.68 cm), with preserved contour, shape, and mediastinal line. The echotexture was mixed, characterized by echogenic lines alternating with hypoechogenic areas distributed throughout the testicular parenchyma. The echogenic lines converged toward the testicular mediastinum, forming a pattern consistent with the arrangement of the seminiferous tubules (Figure 2A). The left testis was located in the inguinal region, between the skin and the abdominal muscle, with preserved contour and shape. However, it was considerably reduced in size (length: 3.3 cm; width: 2.7 cm; thickness: 1.56 cm), appearing more hypoechoic with a homogeneous echotexture, in addition to a well-defined central hyperechoic mediastinum (Figure 2B). Based on these findings, a diagnosis of unilateral cryptorchidism (left inguinal) was established.

### Hormonal and semen analysis

Serum testosterone analysis revealed a concentration of 15,940 pg/mL. The ejaculate obtained by electroejaculation showed a total volume of 1.4 mL and a pH of 8.0. Sperm concentration was 380 × 10^6^ spermatozoa/mL. Kinematic parameters, as well as viability, mitochondrial activity, membrane functionality, and sperm morphology, are presented in Table 1.

**Table 1:** Values of kinetic parameters, membrane integrity, mitochondrial activity, membrane functionality, and sperm morphology of a unilaterally cryptorchid collared peccary *(Pecari tajacu*).

Parameters	Ejaculate	Reference values			Reference
		Mean ± SEM	Min	Max	
Total motility (%)	74	94.2 ± 1.5	82.0	98.0	Santos et al. (2024)
Progressive motility (%)	38	66.8 ± 4.8	41.0	87.0	
Velocity average pathway (µm/s)	49.9	67.6 ± 6.7	42.6	97.7	
Velocity straight line (µm/s)	37.9	47.9 ± 5.7	29.5	78.4	
Velocity curvilinear (µm/s)	100.9	129.8 ± 9.2	84.9	173.3	
Amplitude lateral head (µm)	5.3	6.4 ± 0.3	4.6	7.4	
Beat cross frequency (Hz)	29.9	36.2 ± 1.1	31.4	43.6	
Straightness (%)	75	69.3 ± 2.1	60.0	81.0	
Linearity (%)	39	36.7 ± 2.4	28.0	50.0	
Rapid (%)	66	81.4 ± 4.7	56.0	97.0	
Medium (%)	8	12.6 ± 3.4	1.0	31.0	
Slow (%)	4	2.1 ± 0.5	1.0	5.0	
Static (%)	21	3.7 ± 1.1	1.0	13.0	
Membrane Integrity (%)	69	83.0 ± 3.6	63.0	94.0	
Mitochondrial activity (%)	39	91.8 ± 3.9	65.0	100.0	
Membrane functionality (%)	56	93.7 ± 1.3	83.0	98.0	
Normal morphology (%)	69	67.4 ± 3.3	53.0	82.0	
Detached head (%)	25	1.1 ± 0.4	-	-	Sousa et al. (2013)
Proximal cytoplasmatic droplet (%)	1	2.5 ± 0.7	0.0	8.0	Santos et al. (2024)
Oblique midpiece (%)	3	3.3 ± 0.6	1.0	6.0	
Coiled tail (%)	2	16.4 ± 3.0	8.0	41.0	

### Morphological analysis of the testes

**Figure 3:**
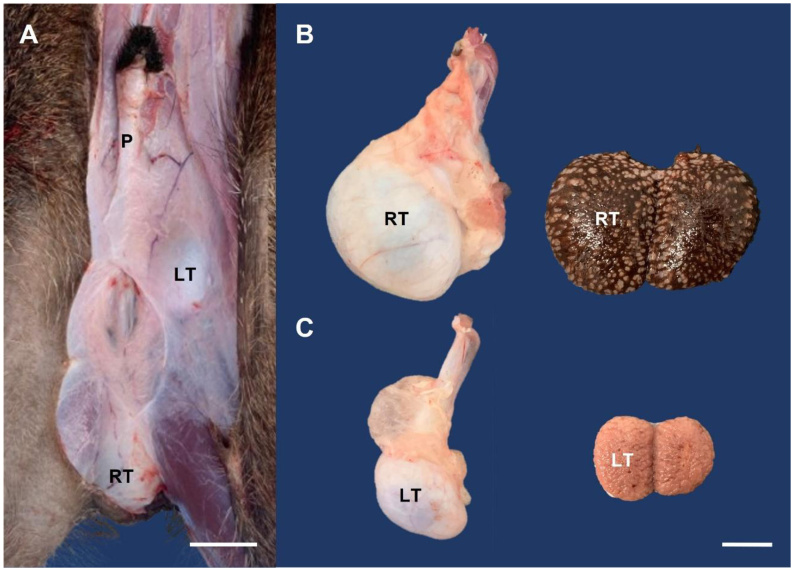
Macroscopic findings of the testes of a collared peccary (*Pecari tajacu*) with unilateral cryptorchidism. (A) Right and left testes *in situ*, with the right testis located within the scrotal sac and the left in the inguinal region. Scale bar: 3 cm; (B) Right testis *ex situ*, showing a heterogeneous parenchyma with dark brown areas (interstitial tissue) and irregularly distributed whitish spots (seminiferous tubules); (C) Left testis *ex situ* (cryptorchid), showing a homogeneous pale pink parenchyma, contrasting with the contralateral testis. Scale bar: 2 cm. Legend: right testis (RT); left testis (LT); penis (P).

Macroscopically, both testes showed preserved shape and contour, surrounded by intact tunics. The right testis measured 6.7 cm in length, 4.8 cm in width, and 3.9 cm in thickness, with a weight of 92.52 g and a volume of 89.05 cm^3^. Its parenchyma exhibited heterogeneous coloration, displaying two distinct tones: a darker shade (dark brown), representing the interstitial tissue, and a lighter (whitish) tone, irregularly distributed across the cut surface, characterized by the coiled arrangement of the seminiferous tubules (Figures 3A, 3B). In contrast, the left testis showed markedly reduced dimensions, measuring 3.6 cm in length, 3.0 cm in width, and 1.8 cm in thickness, with a weight of 25.4 g and a volume of 13.8 cm^3^. Its parenchyma displayed a homogeneous pale pink coloration, clearly contrasting with the appearance of the contralateral testis (Figures 3A, 3C).

**Figure 4:**
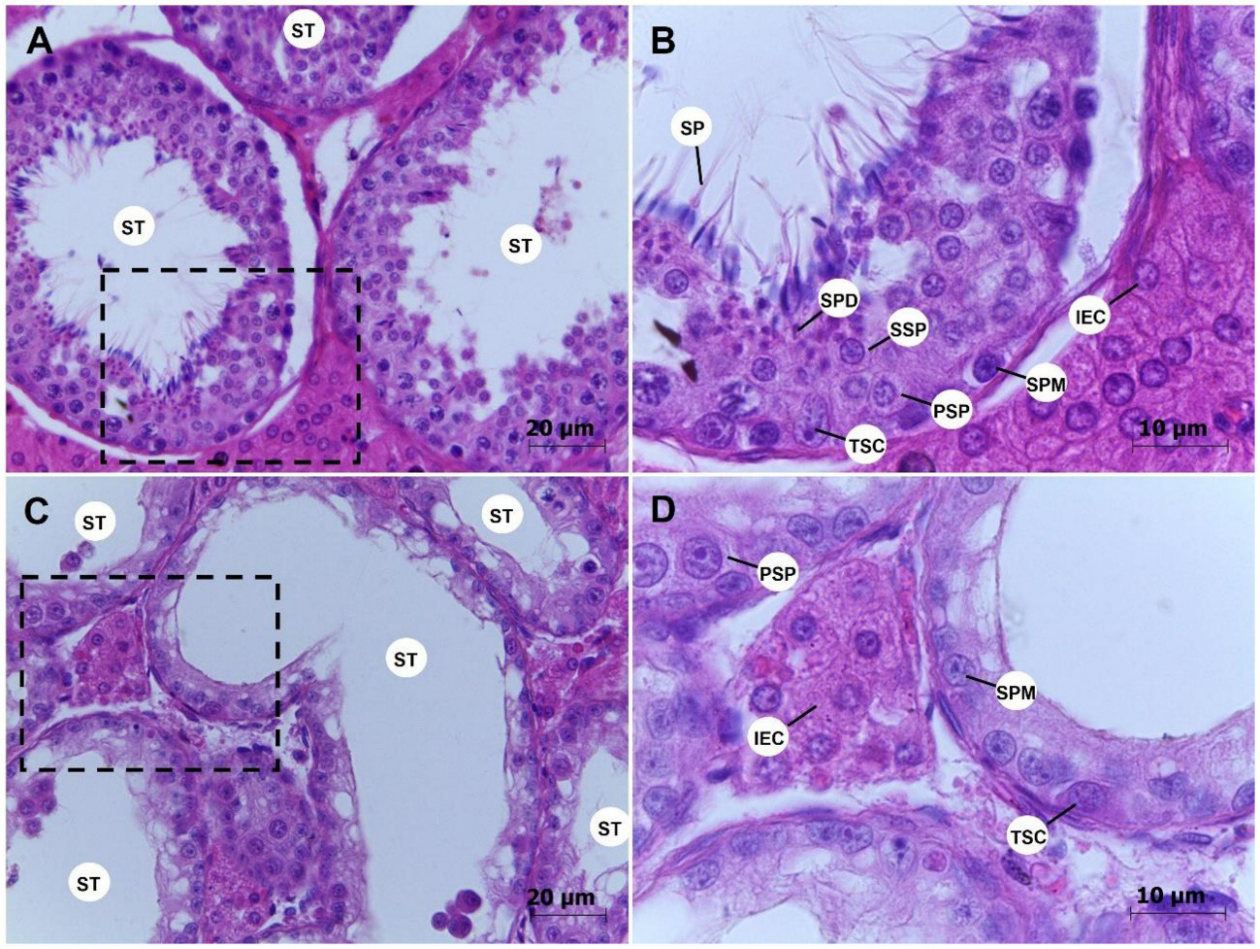
Histological findings of the testes of a collared peccary (*Pecari tajacu*) with unilateral cryptorchidism. (A) Seminiferous tubules of the right testis; (B) Higher magnification of (A), showing the germinal epithelium containing all stages of spermatogenesis, as well as the presence of sustentacular (Sertoli) cells and interstitial endocrine (Leydig) cells; (C) Seminiferous tubules of the left (cryptorchid) testis; (D) Higher magnification of (C), showing a reduced germinal epithelium containing only spermatogonia and primary spermatocytes, with preservation of sustentacular and interstitial cells. Legend: seminiferous tubules (ST); interstitial endocrine cell (IEC); testicular sustentacular cell (TSC); spermatogonium (SPM); primary spermatocyte (PSP); secondary spermatocyte (SSP); spermatid (SPD); sperm (SP). Staining: hematoxylin and eosin.

Histologically, the right testis showed preserved architecture, with seminiferous tubules of regular appearance lined by a germinal epithelium containing all stages of spermatogenesis (spermatogonia, spermatocytes, spermatids, and spermatozoa), in addition to well-distributed supporting cells along the epithelium. The interstitium was highly developed, with abundant interstitial endocrine cells distributed among the tubules, characterizing a functionally and morphologically intact testicular parenchyma (Figures 4A, 4B). In contrast, the left testis exhibited a reduced diameter of the seminiferous tubules and a disorganized and thinned germinal epithelium, containing only spermatogonia and primary spermatocytes, with no evidence of more advanced stages of spermatogenesis. Supporting cells and interstitial cells were present and morphologically preserved. These findings are consistent with testicular degeneration associated with cryptorchidism, with partial impairment of spermatogenesis (Figures 4C, 4D).

## Discussion

The occurrence of unilateral cryptorchidism in this individual may be associated with genetic factors, particularly inbreeding, since the animal belongs to a population with a high degree of relatedness. To date, three cases of cryptorchidism have been identified in this herd: a previous report diagnosed by ultrasonography (Peixoto et al., 2010), an additional case detected during reproductive screening and subsequently removed from the breeding stock, and the present case, indicating a low apparent frequency within the population. The herd currently comprises approximately 139 individuals (55 males and 84 females), although this number has historically fluctuated, reaching more than 300 animals due to experimental use. Although the level of inbreeding was not directly estimated, the population has been maintained for approximately 37 years without the introduction of new breeders, favoring mating among related individuals and a progressive increase in the inbreeding coefficient. This condition is known to increase the expression of deleterious recessive alleles and the occurrence of congenital anomalies, particularly in small or captive populations with restricted genetic diversity (Robinson et al., 2019; Kyriazis et al., 2020; Smeds and Ellegren, 2023). In this context, genetic management strategies such as the introduction of new breeders, control of related matings, and genetic monitoring are essential to increase variability and mitigate the long-term effects of inbreeding (Kinghorn and Kinghorn, 2023).

The clinical diagnosis of cryptorchidism in wild animals represents a challenge, particularly due to difficulties in restraint and anatomical variation among species, especially in those in which testicular descent is seasonal and occurs only during the reproductive period, as described in agoutis (*Dasyprocta* spp.) (Praxedes et al., 2018; Dantas et al., 2021). In such cases, testicular regression may occur, with reduced testicular size, interruption of spermatogenesis, and decreased testosterone levels (Khati and Hammouche, 2021), which complicates the differentiation between a temporary physiological condition and a congenital anomaly. However, in species such as the collared peccary, whose reproductive activity does not exhibit a seasonal pattern (Maia et al., 2019), the absence of a testis from the scrotal sac cannot be attributed to physiological fluctuations.

In the present case, the absence of one testis from the left scrotal sac and the presence of a palpable enlargement in the inguinal region suggested the occurrence of unilateral cryptorchidism. Confirmation was obtained by ultrasonography. The cryptorchid testis showed reduced echogenicity compared with the echogenic pattern expected for the species (Peixoto et al., 2016), reflecting degeneration and alteration of testicular architecture. In contrast, the scrotal testis, although functional, exhibited subtle changes in echotexture compared with the species-specific pattern, in which the parenchyma is typically homogeneous. This difference may be related to compensatory hypertrophy in response to the absence of the contralateral testis, leading to minor structural modifications detectable by ultrasonography.

Morphometric measurements revealed marked testicular asymmetry, with the right testis showing weight, volume, and linear dimensions markedly higher than both the contralateral testis and the mean parameters described for the species (Lara et al., 2019; Maia et al., 2019; Sonner et al., 2004). In contrast, the left cryptorchid testis exhibited a pronounced reduction in volume and dimensions, falling below reference values. This morphometric discrepancy is consistent with testicular degeneration associated with prolonged abdominal retention, in which continuous exposure to body temperature compromises the structural development of the gonadal parenchyma and the maintenance of spermatogenesis (Sharma et al., 2021). Conversely, the hypertrophy observed in the scrotal testis suggests a functional compensatory mechanism, frequently described in cases of unilateral cryptorchidism, whereby the descended testis increases its structural and endocrine activity in order to preserve reproductive function (Dutta et al., 2013).

Although the serum testosterone concentration observed in this individual (15,940 pg/mL) is substantially higher than the range described for clinically normal collared peccaries of the same age group (2,000 – 4,000 pg/mL; Lara et al., 2019; Maia et al., 2019), some important limitations must be considered. Previous studies in this species have primarily focused on seasonal or environmental influences on testosterone levels, with no evidence of significant seasonal variation under certain environmental conditions (Maia et al., 2019). However, to date, there are no studies specifically evaluating circadian variation of testosterone in collared peccaries.

In mammals, testosterone secretion is known to follow a pulsatile and circadian pattern, with fluctuations occurring throughout the day. This phenomenon has been well documented in several species, including domestic pigs (Ellendorff et al., 1975), which are phylogenetically close to peccaries. Therefore, the use of a single blood sample, as performed in the present study and in previous reports for the species, does not allow assessment of possible intra-day hormonal fluctuations, which may influence the interpretation of absolute testosterone values.

Additionally, the laboratory assay employed was validated for domestic pigs, a species phylogenetically close to the collared peccary (family Suidae), but with possible physiological and metabolic differences that may influence the accuracy of hormonal quantification. Although the use of assays validated for related species is a common practice in studies involving wildlife, differences in assay validation, antibody specificity, and calibration standards may limit direct comparisons of absolute testosterone values across studies, including those reported by Lara et al. (2019) and Maia et al. (2019). Therefore, the possibility of analytical interference or overestimation of hormone concentrations cannot be ruled out.

Even so, despite these limitations, the magnitude of the observed elevation, together with the markedly increased morphometric measurements of the scrotal testis, suggests a state of endocrine hyperactivity consistent with a compensatory mechanism. Such a response has been described in cases of unilateral cryptorchidism, in which the descended testis exhibits hypertrophy and increased functional activity in order to maintain reproductive homeostasis (Dutta et al., 2013; Hornakova et al., 2017). Alternatively, the possibility cannot be excluded that this hormonal pattern represents a species-specific physiological characteristic or an atypical condition of this individual, reinforcing the relevance of the present report for expanding knowledge on endocrine–reproductive aspects in the collared peccary.

Semen evaluation revealed a sperm concentration of 380 × 10^6^ spermatozoa/mL, a value lower than that reported for clinically healthy collared peccaries, whose mean concentration is 680.0 ± 68.9 × 10^6^ spermatozoa/mL (Santos et al., 2024). This reduction suggests a partial impact of unilateral cryptorchidism on overall spermatogenic capacity, since only the functional scrotal testis contributed to sperm production. Nevertheless, kinematic and morphological parameters were close to those reported for fertile individuals, with total motility of 74% and normal morphology in 69% of spermatozoa (Table 1), indicating that the descended testis was able to maintain functional spermatogenesis.

This interpretation is supported by the histological findings, in which the right testis exhibited preserved architecture and a complete germinal epithelium, with the presence of all stages of spermatogenesis, whereas the retained left testis showed a thin germinal epithelium and absence of the final stages of the spermatogenic process. In contrast, the reduced values of mitochondrial activity (39%) and plasma membrane functionality (56%) indicate possible limitations in functional fertility. Mitochondrial activity is essential for ATP production, which is required for progressive motility and energy-dependent events such as capacitation and the acrosome reaction (Nesci et al., 2020; Park and Pang, 2021), while plasma membrane integrity ensures sperm viability, protection against oxidative stress, and the ability to fuse with the oocyte (Gualtieri et al., 2021). In this context, even in the presence of morphologically normal spermatozoa, reductions in these functions may compromise fertilization efficiency.

## Conclusion

The results obtained demonstrate that, despite the degeneration observed in the cryptorchid testis, the scrotal testis remained functional, exhibiting compensatory hypertrophy and semen parameters close to the physiological limits described for the species. These findings highlight the need for genetic control and monitoring in breeding and conservation programs for captive collared peccaries, in order to minimize the occurrence of hereditary conditions that may compromise reproductive health and population viability.

## Data Availability

Research data is only available upon request.
